# A critical integrative review of complementary medicine education research: key issues and empirical gaps

**DOI:** 10.1186/s12906-019-2466-z

**Published:** 2019-03-20

**Authors:** Alastair C. Gray, Amie Steel, Jon Adams

**Affiliations:** 1 PhD Candidate Australian Research Centre in Complementary and Integrative Medicine (ARCCIM), Faculty of Health, University of Technology Ultimo, Sydney, NSW Australia; 2 Director New Zealand Operations and Academic Lead, College of Natural Health and Homeopathy, Auckland, New Zealand; 3 Online Academic, Endeavour College of Natural Health Fortitude, Valley, QLD Australia; 4 Senior Research Fellow Australian Research Centre in Complementary and Integrative Medicine Faculty of Health University of Technology, Sydney, NSW Australia; 5 Distinguished Professor of Public Health, ARC Professorial Future Fellow and Director of the Australian Research Centre in Complementary and Integrative Medicine (ARCCIM), Faculty of Health, University of Technology Ultimo, Sydney, NSW Australia; 6 6190 Ardleigh Street, Philadelphia, PA 19138 USA

**Keywords:** Complementary medicine, Education, Andragogy, Challenges, Learning technologies, E-learning, Naturopathy, Education research

## Abstract

**Background:**

Complementary Medicine (CM) continues to thrive across many countries. Closely related to the continuing popularity of CM has been an increased number of enrolments at CM education institutions across the public and private tertiary sectors. Despite the popularity of CM across the globe and growth in CM education/education providers, to date, there has been no critical review of peer-reviewed research examining CM education undertaken. In direct response to this important gap, this paper reports the first critical review of contemporary literature examining CM education research.

**Methods:**

A review was undertaken of research to identify empirical research papers reporting on CM education published between 2005 and 17. The search was conducted in May 2017 and included the search of PubMed and EBSCO (CINAHL, MEDLINE, AMED) for search terms embracing CM and education. Identified studies were evaluated using the STROBE, SRQP and MMAT appraisal tools.

**Results:**

From 9496 identified papers, 18 met the review inclusion criteria (English language, original empirical research data, reporting on the prevalence or nature of the education of CM practitioners), and highlighted four broad issues: CM education provision; the development of educational competencies to develop clinical skills and standards; the application of new educational theory, methods and technology in CM; and future challenges facing CM education. This critical integrative review highlights two key issues of interest and significance for CM educational institutions, CM regulators and researchers, and points to number of significant gaps in this area of research. There is very sporadic coverage of research in CM education. The clear absence of the robust and mature research regarding educational technology and e-learning taking place in medical and or allied health education research is notably absent within CM educational research.

**Conclusion:**

Despite the high levels of CM use in the community, and the thriving nature of CM educational institutions globally, the current evidence evaluating the procedures, effectiveness and outcomes of CM education remains limited on a number of fronts. There is an urgent need to establish a strategic research agenda around this important aspect of health care education with the overarching goal to ensure a well-educated and effective health care workforce.

## Background

The practice, uptake and economics of Complementary Medicine (CM) - a range of therapies, products and approaches to health and illness not traditionally associated with the medical profession or medical curriculum [1] - continues to thrive in many countries [2–7] and concurrently the enrolments at CM education institutions have steadily increased [8, 9]. CM education institutions providing training and qualifications including naturopathy, nutritional medicine, homeopathy, acupuncture, massage therapy and herbal medicine are located across both the public and private tertiary sector in many regions, Australia [10], USA [11], UK [12], Asia [13]. The professionalization of the CM education sector appears to be evolving with continuing professional education, education standards, levels of foundational medical science and higher levels of qualifications emerging in recent years [14–18].

These education institutions face innumerable challenges. These include preparing CM graduates to function as health professionals in a contemporary health system when they apply predominantly traditional principles and concepts [19, 20]. Another challenge is training students in inter-professional care when the focus during training is often on mastering a traditional technique or philosophy [21]. Further challenges involve providing education about evidence-based healthcare when the focus during training is often on learning and applying traditional evidence - defined here as evidence with a long and coherent history of use, well documented in monographs such as materia medica and other texts, mainly inductive in nature, and passed on orally over many generations [22]. This is pertinent in a field that has 700 Cochrane systematic reviews yet one where traditional evidence and knowledge is also highly regarded. Further, providing education on patient-centred care [23], supporting non-traditional students [24, 25] and also gaining funding for and providing education related to perceived non-credible CM modalities in conventional tertiary education settings are challenges [26]. In addition, challenges continue to arise for education leaders both within and beyond CM regarding technological advances and the consequences for students, educators and institutions [27–29]. New developments in healthcare such as e-health/tele-health [30] and a growth in interest in the pedagogy and andragogy of online learning [31, 32] in general, present challenges for educational institutions, professional associations and regulators. Alongside these general educational challenges, faculty resistance to change, the digital divide between students, and between students and faculty, and online readiness for study has been a focus of recent research and discourse in health education [33–36]. More broadly, beyond CM-specific education, tertiary students are increasingly engaging with technology in both their personal and study lives [37, 38] and technology-based learning and teaching in higher education is becoming almost a presumed proposition in many undergraduate courses [39, 40]. CM education is not exempt from such circumstances and there is a necessity for future research on this topic.

In direct contrast to research related to CM practitioner education, there are numerous studies investigating the degree of, and attitudes to CM in conventional medical training [41–43], in biomedical education [44], midwifery [45], pharmacy [46–48] and in nursing training [44, 49–52]. Paradoxically, much of the research regarding CM education relates to its importance and application in nursing education [53], or the experience of integrating naturopathy into nursing educational programs [54], the education of physicians about their patients and CM [55], or addressing the obstacles to success in the implementing of change in science delivery in nursing [56].

The growing CM workforce requires training appropriate to performing evidence-informed, co-ordinated and inter-professional care within the broader health system and developing the evidence-base on this topic will not only aid the CM field but also provide potential insights for health/medical education more broadly [57]. The development of a robust evidence-base on this topic requires a clear understanding of the current landscape. Unfortunately, there has been no critical review of the peer-reviewed research examining CM education to date. In direct response to this important research gap, this paper reports the first critical review of contemporary literature examining a number of key issues across the CM education field.

## Methods

### Aim

The aim of this critical integrative review [58] was to review all published original research found in peer-reviewed literature examining education within higher educational institutions that provide training for CM professionals.

### Method

A database search was undertaken to identify original peer-reviewed literature published from 2005 to 2017 reporting on issues relating to CM education. This date range was chosen to reflect contemporary issues and ensure findings were as pertinent to current practice and policy as possible.

### Search strategy

The search was conducted in May 2017 and included the systematic search of PubMed and EBSCO (CINAHL, MEDLINE, AMED). MESH terms and keywords from related papers were explored to guide the process of selecting search terms, and the process was further refined after referral to a related 2014 review [59]. The first stage was conducted in PubMed. Search A - The search terms embracing CM included, Complementary Therapies, Complementary Medicine, Homeopathy, Naturopathy, Herbal Medicine, Acupuncture, Acupuncture Therapy, Medicine, Chinese Traditional, Massage, Therapy, Soft Tissue, Integrative Medicine, Medicine, Traditional, Holistic Health, Osteopathic Medicine, Manipulation, Chiropractic, Musculoskeletal Manipulations, Physical Therapy Modalities. Filter 2005–2017. (*n* = 258,099). Search B - The search terms embracing education included, education, learning, curriculum, teaching, health occupation students, eLearning, E-Learning, online learning, educational technologies, blended learning. Filter 2005–2017. (*n* = 906,575). A + B Combined (*n = 38,441).* Stage 2 was conducted in EBSCO. The same search terms used in PubMed when entered into EBSCO provided millions of hits, education (*n* = 160 + M) hits, Complementary Therapies (*n* = 629,674) hits and too many potential papers to work. Including ‘eLearning’ and ‘e-learning’ was manageable but these two terms with ‘learning technologies’ made it impossible to proceed. In the process, a review was located which had used similar terms but a different strategy, Milanes 2014 systematic review, *Is a blended learning approach effective for learning in allied health clinicians?* Because of the enormous number of hits using the EBSCO database, and based on this article a revised search method was undertaken for the EBSCO search. Search C - EBSCO Search terms, 1. Online learning OR blended learning or web-based learning, 2. e-learning OR elearning, 3. education* OR curriculum* OR teaching* OR learn*, 4. Combine all (1–3) with AND 5. Complementary Therapies*, 6. Search 4 AND 5 (*n* = 637), 7 Limit to articles from 2005 (*n* = 567 (with duplicates removed)). Search D - This process was completed again searching on the slightly different terminology. Search terms, 1. Online learning OR blended learning or web-based learning, 2. e-learning OR elearning, 3. education* OR curriculum* OR teaching* OR learn*, 4. Combine all (1–3) with AND, 5. Complementary Medicine*, 6. Search 4 AND 5 = 1203, 7. Limit to articles from 2005 = (*n* = 1013 (with duplicates removed)). Stage 3 (Milanes Refined) PubMed. Search E - The search terms Health occupation students OR educational technologies OR teaching OR curriculum AND complementary therapies, filter to last 10 years. Search results = (*n* = 8439). Totals: Search C – *n* = 567, Search D – *n* = 1013, Search E – *n* = 8439. Total Papers *n* = 10,019. Duplicates removed *n* = 523. Grand Total 9496. Manual searching of reference lists of identified papers was also conducted to ensure as full coverage of literature as possible.

### Inclusion and exclusion criteria

Papers written in English, presenting original empirical research data, related to courses where graduates receive a qualification in a CM to a standard accepted by those professions and reporting on the prevalence or nature of the education of CM practitioners in some way were included in the review. Papers reporting conference presentations, or studies relating to how pharmacy, nursing or registered medical professionals are educated regarding their patient behaviours or looking to how they accumulate CPD points in short term CM topics were excluded.

### Search outcomes

**Fig. 1:**
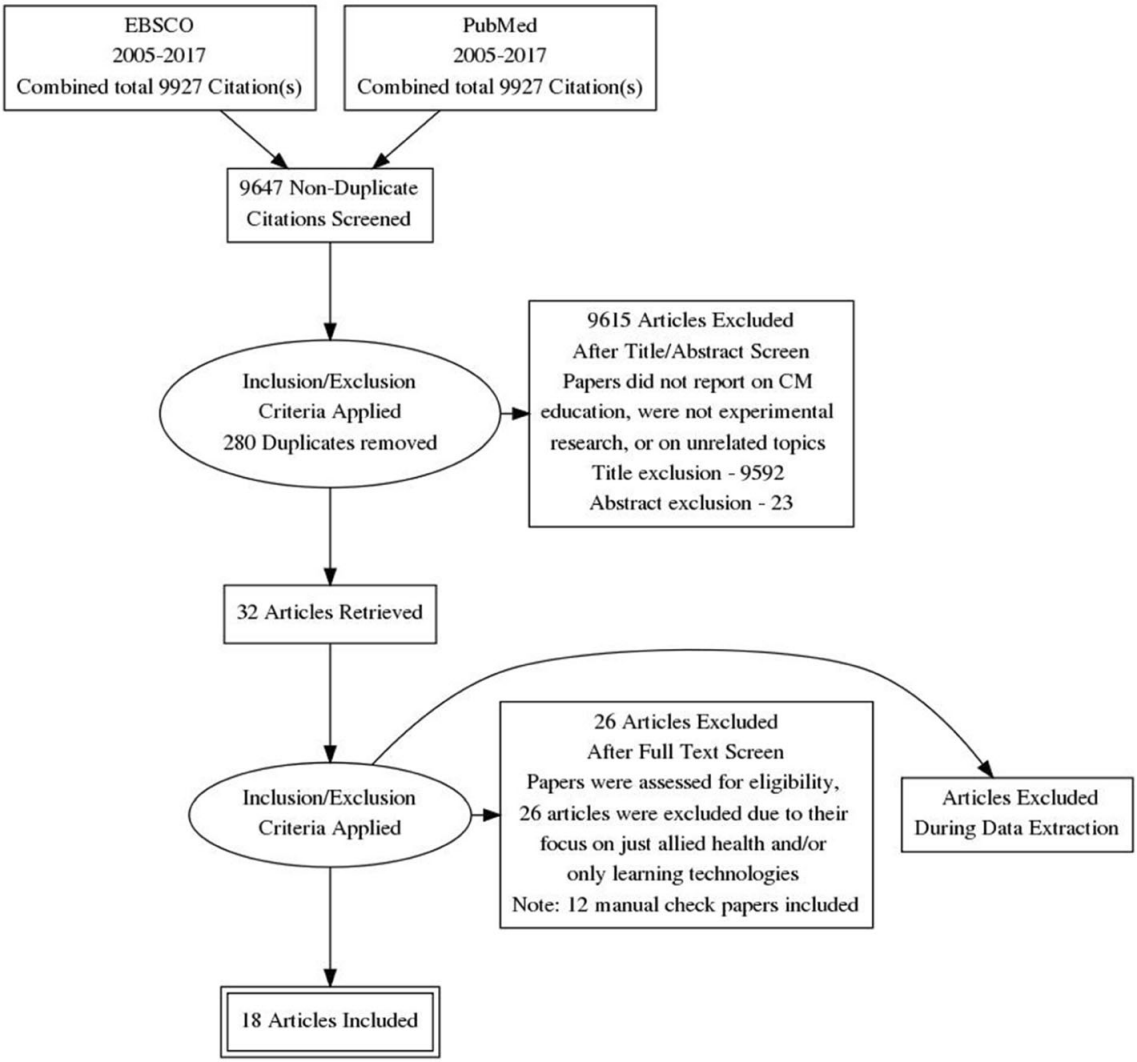
Literature Review Methodology and Selection Process flowchart for articles reporting education and CM (PRISMA Guidelines)

The combined (Complementary Therapies *n* = 420,476 and Education *n* = 102,024) search results (*n* = 9927) were imported into Endnote. Of these, 9895 papers were excluded via title and abstract due to not meeting the inclusion criteria, and all identified duplicates (*n* = 280) were excluded leaving 32 papers. Upon reviewing full papers an additional 26 articles were excluded due to their focus on just allied health and / or only learning technologies with no CM focus; leaving 6 papers. A total of 12 additional papers were identified for review following manual searches. In total, 18 papers were identified for this review. The process undertaken for this review is presented in Fig. 1.

### Critical analysis of included papers

Our critical literature appraisal employed three analytical tools, STROBE [60, 61], SRQR [62] and MMAT [63]. Papers were evaluated for quality and the findings are collated in Table 1.

**Table 1:** STROBE Critical Appraisal Tool for Quantitative Studies

Study	Title Abstract and Introduction			Methods									Results					Discussion and other information					Score/22
	Title Abstract	Background	Objectives	Study design	Setting	Participants	Variables	Data sources	Bias	Study size	Quantitative Variables	Statistical Methods	Participants	Descriptive data	Outcome data	Main results	Other analyses	Key results	Limitations	Interpretation	Geralisability	Funding	Score Out of 22
Forman, L., et al. 2006	x	x	x	x	x	x	x	x		x		x	x	x	x	x		x	x	x			17
Grace, S., et al. 2006	x	x	x	x	x	x	x	x		x		x	x	x	x	x		x	x	x			17
McCabe, P., 2008	x	x		x	x	x	x	x		x			x	x	x	x		x		x			15
Rowe T 2009	x	x		x	x	x	x	x	x	x			x	x	x	x	x	x	x	x	x		18
Steel, A., et al. 2015	x	x	x	x	x	x	x	x	x	x	x	x	x	x	x	x	x	x	x	x			20
Viksveen, P., 2011	x	x	x	x	x	x	x	x		x	x	x	x	x	x	x		x	x	x	x		19
SRQR Critical Appraisal Tool for Qualitative Studies																							
Study	Title & Abstract		Introduction		Methods											Discussion				Other		Score/21	
	Title Abstract	Background	Problem Formation	Purpose of the research question	Qualitative approach and research paradigm	Researcher characteristic and reflexivity	Context	Sampling strategy	Ethics pertaining to human subjects	Data collection methods	Collection instruments and tech	Units of study	Data processing	Data analysis	Techniques to ensure trustworthiness	Synthesis and interpretation	Links to empirical data	Integration with prior work, transferability	Limitations	Conflict of interest	Funding	Score Out of 21	
Chen, Y., et al. 2015	x	x	x	x	x		x	x	x			x	x	x		x	x	x		x		15	
Grant, A., et al. 2012	x	x	x	x	x		x	x	x	x		x	x	x		x	x	x				15	
Viksveen, P., et al. 2015	x	x	x	x	x		x	x	x	x		x	x	x		x	x	x	x			16	
Wardle, J., et al. 2013	x	x	x	x			x	x	x	x		x	x	x		x	x	x	x	x		16	
Wardle, J. and Sarris, J., 2014	x	x	x	x			x	x		x		x	x	x		x	x	x	x	x	x	16	
MMAT Critical Appraisal Tool for Mixed Methods Studies																							
Study	1. QUAL study or QUAL component of an MM study				2. QUAN randomized controlled trial or component of an MM study				3. QUAN nonrandomized study (comparison group) or component of an MM study				4. Descriptive QUAN study (no comparison group) or component of an MM study				5. MM component of an MM study						
	Sources of data relevant to answer the research question	Data analysis relevant to answer the question	Context taken into account in data analysis	Reflexivity of researchers (their influence on findings)	Appropriate randomization (or sequence generation)	Concealment allocation (or blinding)	Complete outcome data	Low dropout rate	Recruitment minimizing bias	Appropriate measurement (validated or standard)	Similar participants in groups (or differences analyzed)	Complete data, high response rate, and appropriate follow-up	Sampling appropriate to answer the research question	Sample representative of the population	Appropriate measurement (validated or standard)	Complete data and high response rate	MM design relevant to answer the research questions	Integration of QUAL and QUAN data and/or results	Consideration of limitations associated with this integration	Score out of 19			
Frenkel, M., et al. 2007	x		x									x	x	x				x		6			
Grace, S., et al. 2007	x		x				x	x		x	x	x	x	x	x			x		11			
Joshi, H., et al. 2013	x		x		x				x	x	x		x		x			x		9			
Long, C., et al. 2014	x		x		x		x	x	x				x	x	x	x		x		11			
Schwartz, J., 2010	x	x	x		x		x		x	x	x	x	x				x	x		12			
Toupin April, K., et al. 2013	x		x		x		x		x	x	x	x	x				x	x	x	12			
Zwickey H et al. 2014	x	x	x		x		x	x	x	x	x	x	x	x	x	x	x	x	x	17			

## Results

Of 9927 identified papers, 18 papers met the review inclusion criteria. An overall synopsis of all papers included in the review incorporated preliminary categorical analysis is outlined in Table 2. The identified studies were conducted in Australia (*n* = 7), the US (*n* = 5), Norway (*n* = 2) and one each from Canada, Taiwan, Israel and India. The research designs reported in the reviewed literature varied widely with quantitative, qualitative and mixed methodologies reported. The quantitative studies selected for review utilized a number of survey design approaches and attracted samples of between 10 and 246 individual participants. The qualitative studies identified employed survey methods [1, 2, 8–14, 18] as well as interviews [1, 6, 11, 15, 16], open essays [2] and focus groups [17]. The spread, focus and identification of themes and topics by CM therapy is represented in Table 2. The naturopathic profession has received most attention from researchers within the international CM education landscape, followed by acupuncture. There are three studies on homeopathy, two studies of chiropractic, and one each of osteopathy, herbal medicine, ayurveda and massage. Six of the included studies focus on a specific class inside of a CM college [1–4, 7, 17], four on academics in CM institutions [6, 12, 16], four studies surveyed members of professional associations [5, 10, 17], and four surveyed College directors [8, 9, 13, 18]. Thematic categorization of the included papers identified four substantive topic areas: (1) CM education provision, (2) the development of educational competencies to develop clinical skills and standards, (3) the application of existing and new educational theory, methods and technology in CM, and (4) future challenges facing CM education.

**Table 2:** Study Characteristics of Included Studies and Thematic Categories (1 CM education provision, 2 The development of educational competencies to develop clinical skills and standards, 3 Application of new educational theory, methods and technology in CM, 3 Future Challenges facing CM education)

	Author/Year	Country	Methods	Data source	Participant recruitment	Key Results/Outcomes reported	Group 1 2 3 4
1	Chen, Y., et al. 2015 [76]	Taiwan	Qualitative. Cross sectional survey. Free form open answers and interviews	Trainees’ survey data were extracted from post-OSCE questionnaires and interviews	Five TCM OSCEs were administered, and the educational backgrounds of the 37 participants were analyzed.	OSCEs can be used in evaluating, teaching, and certifying TCM clinical competencies to improve the quality of TCM practices.	3
2	Forman, L., et al. 2006 [77]	USA	Quantitative. Cross sectional survey	A 27-item questionnaire was distributed to first-through fourth-year osteopathic medical students. Preferred learning methods, current use of computers as an educational tool, and attitudes regarding the role of computers in medical education based on their skill level were evaluated.	246 students (80% of enrolled students) responded to the questionnaire.	Participants in the study were full-time students in the first through fourth years of osteopathic medical school. Students’ opinions of the importance of computer technology in their education is based mainly on their self-assessed technical competency levels. Understanding this dynamic may aid medical educators in the implementation of computer-assisted instruction.	3
3	Frenkel, M., et al. 2007 [70]	Israel	Mixed methods. Observational cross sectional survey.	Pre-course semi-structured questionnaire and an anonymous open essay about students’ experiences with an educational intervention in their final year of study, emphasizing evidence-based learning, patient-centered care, and communication skills with conventional health care providers during 4 academic years, 2001–2005.	62 students were exposed to the educational initiative in integrative medicine to CAM students	CAM practitioners feel better equipped to communicate with conventional health care practitioners after exposure to a structured educational initiative that emphasizes critical thinking, patient-centered care, and communication skills with conventional practitioners.	2
4	Grace, S., et al. 2006 [67]	Australia	Quantitative. Observational cross sectional survey.	45-item questionnaire mailed to members of the Australian Natural Therapists’ Association and the Australian Traditional Medicine Society.	617 responses (22%)	A significant relationship exists between the confidence practitioners had in identifying clients requiring referral and their training in Western medical and CM diagnostic techniques. 32% of respondents reported a lack of confidence in identifying patients requiring referral with the potential to compromise the safety of clients and the effectiveness of practice.	2
5	Grace, S., et al. 2007 [64]	Australia	Mixed Methods. Survey Analysis and Interview	The aim of this study was to compare two CAM curricula: chiropractic and naturopathy. Accredited naturopathy and chiropractic programs in Australia were located. Key learning areas and approaches to clinical training were identified and compared. Course structures and subject/unit descriptions for accredited naturopathic courses were examined via websites where they existed. In addition, Course Co-ordinators, Directors of Study or other appropriate academics/persons from each naturopathic training institution were invited to take part in a short interview (telephone or email) to clarify subject content and course structure and give details of clinical training.	The study found 30 naturopathy courses that conformed to the requirements of either DEST or professional associations. Detailed curricula were available for 17 programs. Interviews, either by telephone or email, were conducted with representatives of 12 training institutions	Chiropractic registration guarantees a uniform level of training for all practitioners. This training was found to comply with accreditation board requirements. The naturopathy courses in the study had elected to comply with the requirements for state government and professional association accreditation, and a level of uniformity was evident amongst the various courses. It is pertinent to note that although both groups of practitioners are entitled to practise as primary contact practitioners, chiropractors and naturopaths had markedly different focuses on medical science training. A review of naturopathy curricula is warranted in the context of uniformity of training for primary contact practitioners.	1
6	Grant, A., et al. 2012 [78]	Australia	Qualitative. Ethno-qualitative research using an ethnographic methodology.	Interviews conducted with ten naturopathy lecturers to investigate reflective approaches to decision making and pedagogy. The scholarly reflections of academic lecturers who taught in the naturopathy program were gathered using interviews and reflective prompts. The approach to the collection and interpretation of data for this investigation was constructivist in epistemology and ethnographic in methodology	Ten individual interviews with key academic lecturers from the disciplinary grouping of Natural and Complementary Medicine (NCM) were undertaken in 2009. Interviews were arranged by email, and semi-structured interviews conducted.	All the naturopathy lecturers interviewed expressed that they had gone through significant changes in their teaching practice as a result of the changes in delivery for the subjects and their exposure to a more involved educational system. This reflective process impacted upon their academic practice as they underwent a process of professional upheaval and reshaping of professional practice.	3
7	Joshi, H., et al. 2013 [75]	India	Mixed Methods (?)	Three educational interventions were applied to a specific subject in Bachelor of Ayurvedic Medicine and Surgery (BAMS) program 2011–2012 and 2012–2013.	Three integrative educational interventions were introduced to develop and evaluate the effectiveness of teaching methods in an Ayurveda curriculum.	The test results in the first experiment showed that the integrative method is comparable with the conventional teaching method. In the second experiment, the test results showed that the integrative method is better than the conventional method. The student feedback showed that all the three methods were perceived to be more interesting than the conventional one. The development of testable integrative teaching methods is possible in the context of Ayurveda education. Students find integrative approaches more interesting than the conventional method.	3
8	Long, C., et al. 2014 [74]	USA	Mixed methods. Cross sectional survey.	A survey to elicit information on the faculty development initiatives was administered via e-mail to 9 program directors. The survey was designed to elicit information in 6 areas: EBP competencies that were developed and adopted; target audiences; size, formats, and hours of training programs; instructional approaches; evaluation methods; and faculty incentives to participate.	All 9 completed the survey, and 8 grantees provided narrative summaries of faculty training outcomes.	The grantees found the following strategies for implementing their programs most useful: assess needs, develop and adopt research literacy and EBP competencies, target early adopters and change leaders, employ best practices in teaching and education, provide meaningful incentives, capitalize on resources provided by grant partners, provide external training opportunities, and garner support from institutional leadership. Instructional approaches varied considerably across grantees. The most common were workshops, online resources, in-person short courses, and in-depth seminar series developed by the grantees. Training programs and workshops are the most useful way to train faculty in evidence based medicine and research literacy.	2
9	McCabe, P., 2008 [67]	Australia	Quantitative. Observational study. Survey	Survey of 43 Australian providers of naturopathy and WHM education. Information sourced from the public record revealed that these providers collectively offered 104 courses in naturopathy and WHM.	Of the 43 providers, 29 valid questionnaires were returned, representing 33 campuses across Australia—a 70.2% response rate by campus.	Educational standards vary widely, with some practitioners not likely to be adequately prepared for practice. There is a need for better integration of complementary care with mainstream healthcare, and education in CM needs to be at least to the level of a bachelor degree.	2
10	Rowe, T. 2009 [66]	USA	Quantitative. Observational cross sectional survey.	Three separate surveys targeted at homeopathic students, homeopathic faculty and homeopathic school directors. It consisted of 40 questions	91.5% of respondents completed the survey. School Director Survey, 20. Teacher Survey, 48. Student Survey, 88.	Homeopathic Schools and Training Programs currently in the United States: 29. Homeopathic Teachers in the United States: 250. Homeopathic Students Currently Enrolled in the United States: 1080.	1
11	Schwartz, J., 2010 [79]	USA	Mixed methods. Observational cross sectional survey and interviews	A survey of faculty teaching at schools in three CM fields and followed up with additional interviews.	NA	Acupuncture, chiropractic, and massage faculty lack awareness of the capabilities of online education and the elements of good online learning, with the perception that what they teach cannot be taught online because of its kinesthetic requirements. The faculty hold this perception in spite of the success of medical science and related health care fields in the online environment, and they do not seem to separate the kinesthetic from the didactic.	3
12	Steel, A., et al. 2015 [72]	Australia	Quantitative. Cross-sectional online survey	The survey included items examining respondent attitudes and beliefs about research, personal research experience, and future intended research activity. Statistical analysis determined descriptive frequencies. Backwards stepwise logistic regression was used to identify characteristics of faculty interested in enrolling in a higher degree by research (HDR).	The survey was completed by 202 of 389 academic and operational staff conducted at a dual sector private CM education institution in Australia.	Respondents perceived research as important to their personal professional goals (86.0%) although confidence in being able to undertake research was less common (56.5%). The perceived importance of publication of research to the respondents’ personal professional goals was also notably high (80.0%) although confidence in their own ability to produce research publications was lower (52.9%).	2
13	Toupin April, K., et al. 2013 [71]	Canada	Mixed methods. Observational cross sectional survey and interviews	A two-phase study consisting of an electronic survey and subsequent semi-structured telephone interviews conducted with curriculum/program directors in regulated Canadian CAM schools. Questions assessed the extent of the research, evidence-based health care, IPC training and continuing education, as well as the C/P directors’ perceptions about the training. Descriptive statistics were used to describe the schools’, curriculum’s and the C/P directors’ characteristics. Content analysis was conducted on the interview material.	28 C/P directors replied to the survey and 11 were interviewed, representing chiropractic, naturopathy, acupuncture and massage therapy schools.	Future CM providers should understand research findings and be able to rely on high quality research and to communicate with conventional care providers as well as to engage in continuing education. Limited length of the curriculum was one of the barriers to such improvements.	2
14	Viksveen, P., 2011 [65]	Norway	Quantitative. Cross sectional survey	Cross sectional survey of current homeopathy undergraduate education in Europe in 2008. Data from 145 (94.8%) out of 153 identified courses were collected. Eighty-five (55.6%) responded to a questionnaire survey. For others some data was extracted from their websites. Only data from the questionnaire survey is used for the main analysis.	Data from 145 (94.8%) out of 153 identified courses were collected. Eighty-five (55.6%) responded to a questionnaire survey plus data from websites.	The average course had 47 enrolled students and 142 graduates, lasted 3.6 years part-time. Of 85 courses most had entry requirements and provided medical education (*N* = 48) or required students to obtain this competence elsewhere (*N* = 33). Average teaching hours were 992 overall, with 555 for homeopathy. Four of five courses were recognised/accredited. Recognised/accredited part-time courses lasted significantly longer than nonrecognised/non-accredited courses, and offered significantly larger numbers of teaching hours in homeopathy. 6500 students were enrolled. 21,000 had graduated from 153 identified European undergraduate homeopathy courses.	1
15	Viksveen, P., et al. 2012 [68]	Norway	Qualitative. Interview	A qualitative study based on grounded theory methodology involving telephone interviews with 17 educators from different schools in 10 European countries. It used constant/simultaneous comparison and analysis to develop categories and properties of educational needs and theoretical constructs and to describe behaviour and social processes. The main questions asked of subjects were “What do you think is necessary in order to educate and train a competent homeopath?” and “How would you define a competent homeopath?”	Telephone interviews with 17 educators from different schools in 10 European countries	The educators defined a competent homeopath as a professional who, through her knowledge and skills together with an awareness of her bounds of competence, is able to help her patients in the best way possible. This is achieved through the processes of study and self-development, and is supported by a set of basic resources. Becoming and being a competent homeopath is underpinned by a set of basic attitudes.	2
16	Wardle, J., et al. 2013 [80]	Australia	Qualitative. Interview	Semi-structured interviews were conducted with 20 naturopaths practising in Australia to explore current perceived challenges in the naturopathic profession in Australia.	20 naturopaths practicing in Australia	Grassroots naturopaths identify a number of challenges that may have significant impacts on the quality, effectiveness and safety of naturopathic care. Given the increasingly mainstream role that naturopaths are playing in the healthcare system in Australia, it is imperative that some of the issues of concern raised by naturopaths receive appropriate policy focus. This may include the development of appropriate regulatory regimes and the development of minimum standards of practice and education that value traditional naturopathic principles and philosophies, as well as ensuring ethical and effective clinical practice.	4
17	Wardle, J. and Sarris, J., 2014 [17]	Australia	Qualitative. Focus groups	Focus groups conducted with current and recent students of 4-year naturopathic degree programs to ascertain how they interact with clinical teaching materials, and their perceptions and attitudes towards teaching materials in naturopathic education.	A total of 24 students and recent graduates participated in the focus groups.	Naturopathic students have a complex and critical relationship with their learning materials. Although naturopathic practice is often defined by traditional evidence, students want information that both supports and is critical of traditional naturopathic practices, and focuses heavily on evidence-based medicine. Students remain largely ambivalent about new teaching technologies and would prefer that these develop organically as an evolution from printed materials, rather than depart from dramatically and radically from these previously established materials.	3
18	Zwickey H et al. 2014 [73]	USA	Mixed methods. Survey and interview	An electronic survey was administered to principal investigators of the nine R25 education grants. The survey consisted of 36 closed- and open-ended questions. Follow- up questions were sent via email to clarify responses as needed. Data were compiled for review and content was analyzed for common themes among institutions. A qualitative analysis was performed using three independent reviewers. This team identified the most successful strategies that the individual institutions used, in addition to the most substantial challenges they encountered.	Nine R25-funded CAM colleges	While each institution designed approaches suitable for its own research culture, the guiding principles were similar and the need to develop evidence-informed skills and knowledge was important to help students and faculty to critically appraise evidence and then use that evidence to guide their clinical practice. These nine CAM institutions faced multiple challenges and developed similar and dissimilar strategies for success. An enriched, EBM-infused CAM curriculum can better prepare future CAM practitioners for communicating effectively with their conventional medicine colleagues. Practitioners in the twenty-first century will need to understand how research and evidence-based practice are related and support one another in order to truly bring about optimal patient care.	2

### CM education provision

The review identified three papers that reported a simple description of educational provision in an area of CM. One study compared naturopathy and chiropractic curricular in Australia. Course structures and subject unit descriptions for accredited naturopathic courses were examined from websites where they existed and in some instances short follow-up interviews were conducted. This study reported the percentage of curriculum devoted to medical sciences and clinical training whereby it was found that on average, chiropractic courses allocated 45.9% of their curricula to medical sciences, whereas university-based naturopathy courses allocated 26.2% to medical science and non-university naturopathy courses allocated 23.1% [64]. Another study reported on the scope of education provision in homeopathy and examined the preponderance of accredited full-time and part-time courses and accredited and non-accredited courses in Europe. This cross-sectional survey of 85 homeopathy education providers found an average of 47 enrolled students and 142 graduates in these generally small schools. Course duration lasted on average 3.6 years part-time, less than half had entry requirements, provided any medical science education or required students to obtain medical science tuition elsewhere. Average teaching hours at surveyed schools were 992 overall, with 555 h devoted to didactic homeopathy study, while the rest focused on clinical training [65]. A similar 2009 study focused on the demographics, satisfaction, challenges and expectations of homeopathy students, teachers and school administrators in North America. The study consisted of three separate surveys targeted at homeopathy students, faculty and school directors consisting of 40 questions with a 91.5% completion rate [66]. It was found that there were 29 homeopathy schools, with 250 teachers and 1080 students currently enrolled in the United States. Programs varied considerably in length; however the average program (670 h) was barely sufficient to meet the minimum standards for homeopathic certification. Homeopathy teachers tend to be older than both homeopathy students or practitioners. The average age of students is 54.3 years old. Although the vast majority of students are female (90%) and practitioners are female (75%), males are much more common as teachers (43.5%) and school directors (45%). As with homeopathy students, practitioners, and teachers, homeopathy school directors are nearly all Caucasian (85%). An important conclusion was that education in homeopathy in the United States has largely remained stagnant in the last 10 years. Although many new schools have been formed, many have closed. It was not speculated as to the cause.

### The development of educational competencies to develop clinical skills and standards

Eight papers from the review focused on improving education and clinical skills in CM. One study reporting findings from 43 education providers of naturopathy and western herbal medicine in Australia found educational standards varied widely, including unsustainable variations in award types, contact hours, clinical education, length of courses and course content with some practitioners unlikely to be trained to professional standards. This study found a need for better integration of complementary care with mainstream healthcare necessitating education to rise to the level of a bachelor degree [67]. The development or application of learning competencies was a focus of these eight papers. Competencies and competency models refer to how the knowledge, skills, and abilities required by these standards are structured. In a study focussing on the skills, knowledge, attributes and competencies of homeopaths and homeopathy education provision, telephone interviews with 17 educators from different schools in 10 European countries were conducted [68]. This qualitative study used constant/simultaneous comparison and analysis to develop categories and properties of educational needs and theoretical constructs and to describe behaviour and social processes and showed educators define a competent homeopath as a professional able to help patients in the best way possible. It was found that course providers and teachers required the competency to be student-centred, and students and homeopaths to be patient-centred [68]. In an Australian study, CM practitioners were reported as having a low level of confidence in identifying clients requiring referral to registered health practitioners, despite the reported high frequency of educational training in, and use of, Western and CM diagnostic techniques [69].

Two identified papers focused on teaching aspects of practitioner communication skills and the integration of complementary and conventional medicine in CM schools. Using a pre-course ‘semi-structured questionnaire’ plus surveys after an educational intervention, 62 students in Israel reported on how the communication gap with conventional physicians and CM practitioners could be improved [70]. This study found that CM practitioners perceived themselves as better equipped to communicate with conventional health care practitioners when critical thinking, patient-centered care, and communicating skills were emphasized in their course of undergraduate study [70]. In addition, a Canadian study published findings derived from 28 directors of colleges of CM. The author reported that student’s ability to understand research findings, to rely on high quality research and to engage in continuing education was important in communicating with conventional care providers [71].

Meanwhile, the need for schools to adopt research literacy and evidence based practice competencies was the focus of three papers. One study that examined the attitudes towards research and scholarly activity of 202 faculty academics in an Australian CM college reported low confidence in undertaking research [72]. Respondents in this Australian study perceived research as important to their personal professional goals (86.0%) although confidence in being able to undertake research was less common (56.5%). The perceived importance of publication of research to the respondents’ personal professional goals was also notably high (80.0%) although confidence in their own ability to produce research publications was lower (52.9%) [72]. Another study conducted in the US examined the approaches of 9 CM colleges to develop evidence-informed skills and knowledge with the aim of developing both students and faculty to critically appraise evidence and then employ that evidence to guide clinical practice [73]. This study found that in developing the framework for their educational programs, educational institutions used strategies that were viewed critical for success, including making them multifaceted and unique to their specific institutional needs. It was found that these strategies, in conjunction with existing instructional approaches, were of practical use in other CM and non-CM academic environments where administrators were considering the introduction of research literacy and EBP competencies into their curricula. Training programs and workshops were found to be the most useful way to train faculty in evidence based medicine and research literacy [74]. Finally, one reviewed paper reported on the educational competencies and institutional teaching strategies that had been developed and implemented to enhance research literacy at all nine R25-funded CM institutions in the US [73]. This study found that while each institution designed approaches suitable for its own research culture, the guiding principles were similar across all, and the need to develop evidence-informed skills and knowledge was important to help students and faculty to critically appraise evidence and then use that evidence to guide their clinical practice. The strategies adopted by these institutions included a need for course content to be conducive to reinforcing EBM competencies using spiral learning strategies, and that faculty were willing to learn and teach EBM skills [73].

### Application of existing and new educational theory, methods and technology in CM

The changing role of the trainer/lecturer in didactic and clinical subjects, the application of existing and new educational theory and problem-based learning within the context of CM curricula in bachelor and medical college programs, as well as the growing use of learning technologies was highlighted by six papers included in the review. In one study three educational interventions testing new teaching methods were introduced in an ayurveda program [75]. The instructional methods that were evaluated were an integrative module on cardiovascular physiology, case-stimulated learning and classroom small group discussion with findings showing the development of testable integrative teaching methods is possible in the context of Ayurveda education [75]. In contrast, findings from an educational intervention, the implementation of an objective structured clinical examination (OSCE) model as well as a patient-centered training approach within traditional Chinese medicine (TCM) practitioner education in one Taiwanese medical school, found this examination approach effective in evaluating, teaching, and certifying TCM clinical competencies to improve the quality of TCM practices. In this study the training program subjects included TCM internal medicine, TCM genecology, TCM paediatrics, TCM dietetics, acupuncture, TCM orthopaedics, and traumatology [76].

When it comes to resources and the use of technologies, Wardle’s 2014 study used focus groups with current and recent students of 4-year naturopathic degree programs in Australia to ascertain how they interact with clinical teaching materials, and their perceptions and attitudes towards teaching materials in naturopathic education. This study described a desire among naturopathy students for existing curriculum to focus on evidence-based approaches and information that both supported and was critical of traditional naturopathic practices. These students remained largely ambivalent about new teaching technologies and preferred that these develop organically as an evolution from printed materials, rather than depart dramatically and radically from these previously established materials [17]. CM student’s preferred learning methods are often based on levels of computer skills and experience, their current use of computers as an educational tool, and attitudes regarding the role of computers in medical education according to a cross sectional survey study from a 27-item questionnaire distributed to 1–4-year Osteopathic medical students in the US [77]. One ethnographic study based on interviews conducted in the Australian university system with Naturopathic Faculty found an openness to the utilization of a number of technologies for flexible learning, including wikis, podcasts and synchronous audio-based online interactions [78]. In contrast, another study in the US found acupuncture, chiropractic, and massage therapy faculty lacked awareness of the capabilities of online education and the elements of good online learning and described a perception that what they taught could not be taught online because of its hands-on kinaesthetic requirements such as palpation [79].

### Future challenges facing CM education

Lastly, one paper included in our review identified some of the challenges ahead for the Australian naturopathic profession including naturopathic education, the changing student body in naturopathic education, naturopathic student expectations, and the growing tension between traditional and scientific evidence [80]. This study, involving semi-structured interviews with 20 naturopaths, found that participants articulated a paradox whereby on the one hand, they supported the teaching of increased levels of biomedical sciences in naturopathic education, yet also complained of the trend of contemporary naturopathic education to “become more scientific” – a trend they attributed to their desire for the discipline to be “accepted in the university sector”. The participants claimed that such a development would be undertaken at the expense of the philosophical underpinnings of the profession. The authors found the continued development of minimum standards of practice and education that value traditional naturopathic principles and philosophies in tandem with the development of appropriate regulatory regimes, was vital in ensuring continued ethical and effective clinical practice.

### Quality of papers

Based on the STROBE reporting guidelines [60] the quantitative papers included in this study, while rich in design, descriptive data and discussion of results exhibited a broad weakness in stating clear objectives. In addition, statements and acknowledgement of bias were mostly absent. Other elements commonly missing from these papers were descriptions of statistical methods and generalisability leaving a general impression of low quality among the included papers. Based on the SRQR [62] tool for evaluating qualitative studies, all selected papers omitted a discussion on the qualitative approach and research paradigm used. A description of researcher characteristics and reflexivity, and techniques to enhance trustworthiness and credibility of data analysis were mostly missing. In addition, potential sources of influence or perceived influence on study conduct and conclusions and how these were managed were also under-reported across this collected literature. In addition, a lack of reporting on sources of funding and other support, the role of funders in data collection, interpretation, and write-up were other weaknesses identified. The application of the MMAT critical appraisal tool for the mixed methods studies [63] in this instance found all papers used, included and reported appropriate sources of data relevant to answer the research question, took into account the context in data analysis, used appropriate sampling to answer the research question, and integrated qualitative and quantitative data and/or results. On the other hand, only some papers applied features of the tool such as data analysis relevant to answer the research question, and only a few reported on complete outcome data, or dropout rate, reported on recruitment minimizing bias and appropriate follow-up, used appropriate randomization, appropriate measurement, sample representative of the population, or appropriate measurement. No papers reported on the reflexivity of researchers, nor concealment allocation, and only a few reported on the mixed method design relevant to answer the research questions, integrated the mixed qualitative and quantitative data and results nor took into consideration any limitations associated with this integration, leaving an overall impression of poor quality.

## Discussion

This critical integrative review highlights two key issues and large current empirical gaps. Firstly, given the growing popularity of CM and as a consequence the growth in CM education, there is very sporadic coverage of research in the CM education field. Across the 18 included papers, research from 7 countries is represented with 4 of those countries having only one identified relevant paper. In addition, the quantity and quality of available evidence invariably relates to disparate, random and unrelated parts of CM education philosophy and practice. Our review findings highlight that much of the research is now relatively dated [81]. In addition, there is extreme diversity in the represented professions and ultimately the quality of papers. Many papers were excluded due to inconsistencies between title, abstract and findings [82]. Some papers were relevant but not published in peer reviewed journals and thus excluded; highlighting how in a maturing field there is a need to publish in both professional industry journals and the peer reviewed literature. One such example was the result of a survey of ‘profession-wide’ educational acupuncture institutions in the US as well as an extensive literature review, subject matter expert interviews, community discussions, strategic planning, analysis, and evaluation, that called for the development of educational competencies [83]. Others examples were the Survey on Inter-institutional and Interprofessional Relationships of Accredited Complementary and Alternative Medicine Schools and Consortium of Academic Health Centers for Integrative Medicine Programs [84], the National Education Dialogue to Advance Integrated Health Care: Creating Common Ground [84], the Project for Inter- Institutional Education Relationships [85]: Examples of Real Life Inter-Institutional Collaborations [84], and the Credentialing Licensed Acupuncture and Oriental Medicine Professionals for Practice in Healthcare Organizations: An Overview and Guidance for Hospital Administrators, Acupuncturists and Educators [84].

Our review identified that whilst educational standards and practices were considered within original research articles related to CM, this was mostly as part of the contextual discussion of findings of related but not directly relevant CM research. This pattern was observed both in the grey literature [86, 87] and peer-reviewed publications [8, 15, 54, 67, 88–99]. One striking example of research emphasizing information related to CM education but collected in other settings, is by Wardle and colleagues in which practicing naturopaths were interviewed regarding multiple issues including the public misconception of the role of naturopathic medicine, the devaluation of naturopathic philosophy as a core component of naturopathic practice, the pressure to move towards an evidence-based medicine model focused on product prescription, as well as naturopathic education. In this paper, much of the data collected related to CM education but came from a broader research question and sample than research which focuses specifically on education and relevant stakeholders. Similarly, in Steel’s 2011 article, 12 naturopaths in current clinical practice were interviewed on the sources of information used in clinical practice, and the participants’ perceptions of these sources. This elicited comments about naturopathic education as well as concluding comments by the authors in relation to naturopathic education [89].

Another major finding from this review is that the robust and mature research exploring educational technology and e-learning that is taking place in medical and or allied health (nursing, midwifery, pharmacy) education research is clearly absent within the CM educational research field. Research within conventional medical and allied health education has explored the value of educational technology in place of traditional face-to-face delivery or within clinical training [100, 101]. Moreover, there is also now substantial research examining the culture change for stakeholders in medical and allied health education, with qualitative research drawing on the results of surveys reporting of student and staff characteristics for developing faculty, or reporting on digital literacy and other academic processes as a consequence of e-learning [102, 103]. In addition, many case studies of educational interventions have been published using some aspect of e-learning in medical or health services training [104]. Finally there are many original research papers examining the challenges facing medical education due to the clear trends of changing student behaviour, often as a result of the use of learning technologies [105–107]. Further, there are numerous studies exploring more effective delivery methods, and the development of critical thinking [108, 109]. None of these areas of research relating to learning technologies have being reported nor evaluated in CM at present. This highlights that there is a significant discourse relating to andragogy and learning technologies taking place in arenas not too distant from CM education but not within CM practitioner education itself. These findings also highlight that most of the research on CM education is in non-CM environments or in arenas possibly similar to CM, such as nursing or Integrative Medicine but not CM.

### Consequences

As identified in this review, the current evidence evaluating the procedures, effectiveness and outcomes of CM education remains limited in many significant areas despite the high levels of use of CM in the community and the thriving nature of CM educational institutions globally. As a result, there are a number of challenges previously described by commentators [8] which impact on the growth and sustainability of CM education. In particular, the ongoing absence of strategy in CM education research ensures a gap in the available knowledge and contributes to uncertainty for CM education leaders, policy makers and other health professionals as to the needs of employers and the market [8]. Furthermore, our review reveals the current empirical data regarding CM education as affording only a limited, superficial understanding of contemporary CM education highlighting the sporadic spread and apparent scarcity of research in this field. Our research suggests that possibly practitioners and users of CM for so long out of mainstream health care activity in Western societies [3, 110, 111] hold unique values attitudes to health [79]. In addition, it possibly suggests that there is in general a slower adoption of technology, and a stronger culture of resistance to change [78]. Yet it also points to a selective use of technologies as there is growing evidence of innumerable CM consultations taking place online [112, 113]. This relatively low amount of empirical data pertaining to CM research in general may also be explained by the fact that there are few research active CM academics [8] and this is underpinned by a lack of perceived relevance of research in CM educational entities that are for the most part more technical colleges with academics often focused on technical and clinical expertise rather than empirical research activities [72].

### Research opportunities and directions

The findings of this review highlight that there are significant gaps in the existing research examining CM education. There is a need to establish a strategic research agenda in this field. To effectively address these gaps it is important that future research build on a strong understanding of the unique educational environment of CM courses, colleges and universities. A key foundational step to developing a better understanding of the effectiveness of CM education is to more clearly identify current CM educational provision. Building upon a health services research approach, future research is required which examines the characteristics, attitudes, preferences, experiences and motivations of modern CM students. There is an urgent need to understand CM educational institutions geographical location, enrolment patterns, andragogy, their size and scope as well as international similarities and differences. This is particularly important given the as yet largely unexplored and potentially unique characteristics of CM educational institutions and their similarities or differences with other health services education (nursing, pharmacy), the potential size of the CM education market and the numbers of graduates entering CM professions. Alongside this, a closer examination of the use and reliance on technologies, faculty attitudes to technologies and change, the demographics, psychographics and the values of faculty at CM colleges is needed. Moving forward there is a need to understand how changing educational trends relate to CM, if CM educational settings are distinct because of their unique student body, the difference between training CM practitioners and training people about the use of CM, the broad and differing landscape of CM education provision across the world, to what degree are CM educational institutions influenced by the broader trends taking place in education globally? CM education is not immune or separate from the changes taking place in education globally (Massive Open Online Courses (MOOCs), flipped classrooms, constructivist education theories and the implementation of problem based learning [114–119] and this points the way forward for CM education research. Such an examination of CM education must also include the cultural diversity of education provision, local regulations and nuances. A broader knowledge of how health services education informs CM education, the degree to which research and evidence in health services education can be scaled to CM education [120], and how the foundational sciences are taught in CM institutions is also required. It might be beneficial for Colleges to explore strategies to develop faculty in areas such as e-learning technologies, research literacy and evidence-based practice skills, and students in health literacy and information literacy. For this, faculty and administrative champions are needed, as are early adopters and change-leaders. More needs to be known about the sheer breadth of educational provision in CM internationally, the range of award options with courses currently available at undergraduate, and postgraduate level, the relationship between prerequisites, the content of the program and the graduate outcome, as well as pathways include promoting CM-related courses in higher education generally, such as meditation, or somatics. General education courses to benefit all students and entry requirements and education prior to CM training, which may improve science literacy, publication productivity, professional standards. In addition more needs to be known about the financial drivers of for profit private equity educational institutions in the CM field.

### Limitations

These findings can be contextualised within identifiable limitations. Searching literature related to CM can be challenging due to the lack of a consistent international definition. Further, there are many relevant studies, papers and commentaries that are not peer-reviewed and fell outside the scope of this review. There were 12 papers in this review that were identified through manual searching. This possibly highlights that despite research being conducted in this area, papers may not be published in journals which are indexed in commonly searched research databases. Whether this is due to a perception amongst CM-specific or health professional education journals that research in CM education falls outside of their relative scope and prefer to focus on clinical questions or the researchers are not targeting these other journals is not clear. Moreover, the application of three critical appraisal tools created challenges of inclusion and exclusion related to quality. In the case of the SRQR and MMAT, these guidelines were written for pure qualitative and mixed methods research, yet the papers in this review were published in public health and education journals. As such, the structure and content of the included qualitative and mixed methods articles may have been modified to suit the journal style guide and intended audience and the reporting guidelines may have been compromised as a result. For this reason, the low score for some of these articles may be due to reporting omissions of necessity rather than true gaps in methodology. Another limitation identified is that conducting research that crosses national borders comparisons become challenging. There are quite different standards for entry level and practice even between the various professions from country to country. The findings of the article reflect a symptom of wider issues and general statements are necessarily hesitant in this context. Nevertheless, where possible these limitations have been mitigated through attending to systematic review best practice, and as a consequence the relevance and value of the findings presented here for contemporary healthcare education provision should not be minimised.

## Conclusion

Despite the high rates of CM use worldwide and growing interest in CM education, only a sporadic and under-developed body of original research has examined relevant issues to date and there is a need for both a growth in research activity and a clear coordinated research agenda in this important topic area. The significance of growing such a research program around the broad topic of CM education is essential to ensuring an adequately trained and educated CM workforce capable of realising an important role in the broader, coordinated and inter-professional health care system.

## Abbreviations

CM: Complementary Medicine
EBM: Evidence Based Medicine
HREC: Human Research Ethics Committee
MMAT: Mixed Methods Appraisal Tool
MOOC: Massive Open Online Course
OSCE: Objective Structured Clinical Examination
PRISMA: Preferred Reporting Items for Systematic Reviews and Meta-Analyses
SRQR: Standards for Reporting Qualitative Research
STROBE: Strengthening the Reporting of Observational Studies in Epidemiology
TCM: Traditional Chinese Medicine
US: United States
